# Maternal Socioeconomic Factors and Racial/Ethnic Differences in Neonatal Anthropometry †

**DOI:** 10.3390/ijerph17197323

**Published:** 2020-10-07

**Authors:** Calvin Lambert, Jessica L. Gleason, Sarah J. Pugh, Aiyi Liu, Alaina Bever, William A. Grobman, Roger B. Newman, Deborah Wing, Nicole M. Gerlanc, Fasil Tekola-Ayele, Katherine L. Grantz

**Affiliations:** 1 Epidemiology Branch, Division of Intramural Population Health Research, Eunice Kennedy Shriver National Institute of Child Health and Human Development, National Institutes of Health, Bethesda, MD 20892, USA; clambertjr@gmail.com (C.L.); jessica.gleason@nih.gov (J.L.G.); sap790@gmail.com (S.J.P.); abever16@gmail.com (A.B.); fasil.ayele2@nih.gov (F.T.-A.); 2 Biostatistics and Bioinformatics Branch, Division of Intramural Population Health Research, Eunice Kennedy Shriver National Institute of Child Health and Human Development, National Institutes of Health, Bethesda, MD 20892, USA; liua@mail.nih.gov; 3 Feinberg School of Medicine, Northwestern University, Chicago, IL 60611, USA; w-grobman@northwestern.edu; 4 Department of Obstetrics and Gynecology, Medical University of South Carolina, Charleston, SC 29425, USA; newmanr@musc.edu; 5 Irvine, Obstetrics & Gynecology, School of Medicine, University of California, Orange, CA 92697, USA; deborah.wing@kornferry.com; 6 Fountain Valley Regional Hospital and Medical Center, Fountain Valley, CA 92708, USA; 7 The Prospective Group, Arlington, VA 22209, USA; nicole.gerlanc@nih.gov

**Keywords:** abdominal circumference, biparietal diameter, birthweight, disparities, fetal growth, head circumference, neonatal length, singletons, socioeconomic status

## Abstract

Disparities in birthweight by maternal race/ethnicity are commonly observed. It is unclear to what extent these disparities are correlates of individual socioeconomic factors. In a prospective cohort of 1645 low-risk singleton pregnancies included in the NICHD Fetal Growth Study (2009–2013), neonatal anthropometry was measured by trained personnel using a standard protocol. Socioeconomic characteristics included employment status, marital status, health insurance, annual income, and education. Separate adjusted generalized linear models were fit to both test the effect of race/ethnicity and the interaction of race/ethnicity and socioeconomic characteristics on neonatal anthropometry. Mean infant birthweight, length, head circumference, and abdominal circumference all differed by race/ethnicity (*p <* 0.001). We observed no statistically significant interactions between race/ethnicity and full-time employment/student status, marital status, insurance, or education in association with birthweight, neonatal exam weight, length, or head or abdominal circumference at examination. The interaction between income and race/ethnicity was significant only for abdominal circumference (*p =* 0.027), with no other significant interactions for other growth parameters, suggesting that racial/ethnic differences in neonatal anthropometry did not vary by individual socioeconomic factors in low-risk women. Our results do not preclude structural factors, such as lifetime exposure to poverty, as an explanation for racial/ethnic disparities.

## 1. Introduction

Race/ethnic disparities in pregnancy outcomes continue to be a pressing public health issue [1], and identifying contributing factors to these disparities is an ongoing area of research. The NICHD Fetal Growth Studies–Singletons was a prospective cohort study designed to establish standards of fetal growth and determine the need for racial/ethnic specific standards [2]. Significant racial/ethnic differences in fetal growth, fetal growth velocity, and birthweight were observed even after adjusting for detailed demographic and socioeconomic factors that differed among the groups including insurance, annual income, education, and marital status [3,4]. However, whether race/ethnic differences in birthweight varied by these socioeconomic factors was not directly assessed.

A survey found that both poor (defined as households with incomes less than 100% of the federal poverty level) black, and white women were more likely to deliver low birthweight infants than their counterparts with higher incomes, while poor white women had lower rates of low birthweight, small for gestational age and preterm birth than affluent black women [5]. These findings have been corroborated by a number of other studies [6,7], highlighting the complexity of associations between socioeconomic factors, race, and fetal growth.

A closer investigation into socioeconomic variables in relation to neonatal anthropometry may provide further insight into the clinical implications of birthweight differences across race/ethnic groups, since neonatal body composition, which reflects adiposity [8], is predictive of adult metabolic health and cognitive function [8,9,10,11,12]. Given the low risk status of this study population, our objective was to explore whether the previously observed racial/ethnic differences in neonatal anthropometry and birthweight varied by socioeconomic factors.

## 2. Materials and Methods

This was a secondary analysis of the NICHD Fetal Growth Studies–Singletons, a prospective cohort study across 12 community and perinatal centers between July 2009 and January 2013 [13]. The primary aim of the original NICHD Fetal Growth Study was to establish a standard for normal fetal growth (velocity) and size for gestational age in the U.S. population. To achieve this aim, the study recruited low-risk singleton pregnancies across four self-identified race/ethnic groups; non-Hispanic White, non-Hispanic Black, Hispanic, and Asian/Pacific Islander. Inclusion criteria included maternal age 18–40 years; pre-gravid body mass index (BMI) 19.0–29.9 kg/m^2^ calculated from recalled pre-pregnancy weight and height; viable singleton pregnancy between 8 weeks 0 days (8w0d) to 13 weeks 6 days with gestational age consistent with the last menstrual period dating within a prescribed range per screening sonogram. Women with prior adverse pregnancy outcomes, history of chronic disease, conception using medical drugs or assisted reproductive technology, cigarette smoking, illicit drug use or intake of ≥1 alcoholic drinks per day were excluded, as previously described [2,4]. For the Fetal Growth Standard, we also excluded women with pregnancy complications, including preterm delivery (<37 weeks), gestational diabetes, and hypertensive diseases, as well as neonatal conditions including congenital anomalies and death [2]. Ethical approval was obtained from all participating sites (institutional review board #09-CH-N152, approved December 2009) and women gave informed consent (ClinicalTrials.gov Identifier: NCT00912132).

At enrollment, research nurses conducted in-person interviews to obtain detailed demographic and health characteristics. Women were followed from enrollment through delivery. Birthweight at delivery was abstracted from labor and delivery hospital records by trained research personnel. Standardized neonatal anthropometric measures were usually obtained within 1 to 3 days after birth (median (interquartile range) 1 (1,2)) depending on neonatal condition and timing of discharge. Trained research nurses completed the exam assisted by a second person who helped hold the infant in position for measurements of weight (neonatal weight), length, head circumference, and abdominal circumference. Weight was measured using an electronic infant or beam balance scale and was recorded in grams (g) [14]. Length in centimeters (cm) was measured, with the infant lying flat on their back, as the distance from the soles of the feet to the top of the head using a SECA 416 Infantometer (SECA, Hamburg, Germany) [15,16]. Head circumference was taken with a tape measure as the distance from the forehead above the eyebrows, posteriorly around to the maximum protrusion of the occiput and back to the starting point [17]. The abdominal circumference was determined by placing the tape on the abdomen above the umbilicus and perpendicular to the long mid-axis of the trunk [17,18,19,20]. Circumference measurements were made to the nearest 0.1 cm and all measurements were taken at least twice. A third measurement was included if any of the first two measurements differed more than the expected technical error rate for that measurement [21,22,23,24,25].

Trained research personnel abstracted demographic data, antenatal history, and labor, delivery and neonatal course and outcomes from the prenatal record, labor and delivery summary, and hospital and neonatal records.

### Analysis

Baseline maternal characteristics, infant sex, and socioeconomic factors were compared by self-reported race/ethnicity. Significance was determined using $\chi^{2}$ or one-way ANOVA for categorical and continuous data, respectively. Separate generalized linear models were used to test the potential modifying effect of race/ethnicity and the combination of race/ethnicity and socioeconomic characteristics on neonatal anthropometry. We tested this effect by including two-way interaction terms (socioeconomic factor*race/ethnicity) for each socioeconomic factor. Both one-way models and two-way interaction models were adjusted for days from birth to measurement (except birthweight), infant sex (male/female), maternal characteristics: age, height, pre-gravid weight, parity, and other maternal socioeconomic factors: full-time employment/student status (yes/no), marital status (married or living as married/not), health insurance (private or managed care/other), annual household income (<$30,000, $30,000–39,999, $40,000–49,999, $50,000–74,999, $75,000–99,999, ≥$100,000), and education (<high school, high school or equivalent, some college or associate degree, bachelor’s degree, and master’s or higher degree). All covariates were treated as continuous unless otherwise stated. We did not adjust for gestational age at delivery because it is an intermediate on the path to neonatal anthropometry. Additionally, racial/ethnic differences in fetal growth have previously been shown to be persistent across developmental weeks using these data [2]. Thus, it is unlikely that gestational age at delivery would further confound any observed associations and its inclusion may be more likely to introduce bias. Where self-reported height and weight data were missing (*n* = 1 and *n* = 4 respectively), measured height and weight data were used instead. We used multiple imputation (with 20 imputations) to account for all other missing covariate information; missing income (*n* = 224) and missing marital status (*n* = 2). Least squared means comparisons (Tukey method) were performed where the interaction between race/ethnicity and categorical socioeconomic traits were significant.

All analyses were implemented using SAS (version 9.4, SAS Institute, Inc., Cary, NC, USA). Significance was set at *p* < 0.05 for all analyses.

## 3. Results

There were 1737 women included in the fetal growth standard. For this analysis, we excluded six who refused to continue and 86 without any neonatal anthropometry measurements, for a final analytic sample of 1645.

Table 1 presents maternal and neonatal characteristics across different race/ethnic groups. The majority of women in each race/ethnic group were either full-time employed or students and were married or living with a partner although the percentage was lower for non-Hispanic black women (48%). The majority of non-Hispanic white and Asian/Pacific Islander women had private or managed care plans while non-Hispanic black and Hispanic women utilized other plans for health care access. A large proportion of non-Hispanic black (48.3%) and Hispanic (38.2%) women had income below $30,000 while most non-Hispanic white and Asian/Pacific Islander women had income greater than $100,000.

Neonatal anthropometry outcomes were significantly different among racial and ethnic groups when adjusted for covariates (Table 2). In our comparison among race/ethnic groups, the non-Hispanic white group had significantly higher birthweight and neonatal exam weight, as well as greater exam length, and head and abdominal circumference than all other groups, while the non-Hispanic black group had smaller anthropometrics than all the other groups.

Table 3 presents neonatal anthropometric measurements with respect to socioeconomic factors. Neonatal anthropometric measurements varied by maternal marital status, insurance, family income, and education, but not full-time employment/student status. 

**Figure 1:**
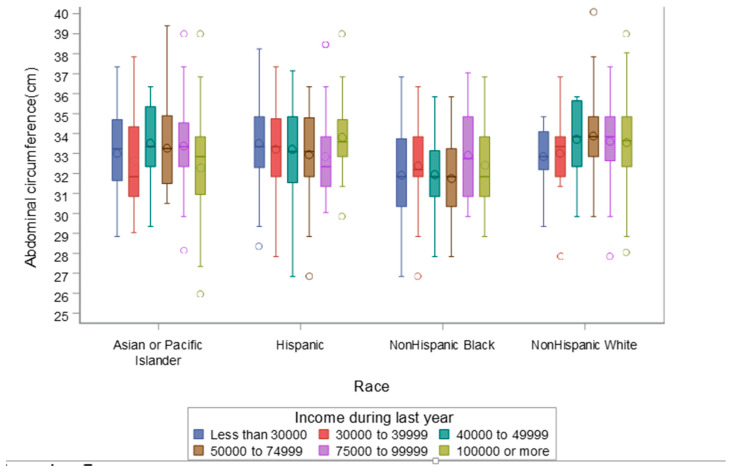
Income level differences in fetal abdominal circumference by race.

There were no significant interactions between race/ethnicity and full-time employment/student status, marital status, insurance, or education in association with birthweight, neonatal weight, length, or head or abdominal circumference at examination (Table 4). The interaction of income with race/ethnicity was significant for abdominal circumference only (*p =* 0.027). There were no significant interactions of income with other neonatal parameters. The relationship between income and abdominal circumference stratified by race/ethnicity is presented in Figure 1. Within non-Hispanic white or Hispanic groups, abdominal circumference did not vary significantly by income category. Within the Asian cohort, abdominal circumference was larger for an income of $75,000 to 99,999 relative to an income of $100,000 or more (*p* = 0.0065). Within the non-Hispanic black group, abdominal circumference was smaller for incomes of less than $30,000 and incomes of $50, 000 to $74, 999, relative to incomes of $75,000 to $99,999 (*p* = 0.0362 and *p* = 0.0187, respectively). Differences among other income categories within the non-Hispanic black group were not significant.

## 4. Discussion

In a racial/ethnic diverse cohort of low risk women, we found that racial and ethnic differences in neonatal anthropometry could not be explained by commonly measured individual socioeconomic factors. Of the growth parameters evaluated, income may have moderated the association between race/ethnicity and abdominal circumference only, and the degree of variability was more prevalent within the black cohort for incomes that differed by more than $60,000.

Our findings highlight the complexity of the interaction between socioeconomic factors and race/ethnicity and are generally consistent with other studies that suggest that socioeconomic differences may not fully account for racial and ethnic disparities in neonatal anthropometry. Frisbie et al. investigated this relationship using the National Maternal and Infant Health Survey (NMIHS), which included a high-risk cohort of pregnant women with varying degrees of prenatal care (inadequate versus adequate as measured by the Kotelchuck Index). Using multivariable analysis, they concluded that the net effects of education, income, and receipt of welfare had no significant impact on birthweight overall [26]. Another investigation utilizing data from the Early Childhood Longitudinal Study-Birth Cohort found that education and marital status were not associated with low birthweight across race/ethnic groups, and poverty status was only associated with low birthweight among US-born Mexican women [27]. In a study of disparities in child and adult health outcomes, Braveman et al. provided evidence of a gradient effect between socioeconomic measures and health outcomes in both non-Hispanic white and black populations, such that health outcomes improve as socioeconomic status increases for both groups [7].

Common across these investigations is speculation that individual socioeconomic factors may be inadequate to explain the complex associations between race/ethnicity and neonatal anthropometry, as suggested by studies of area-level factors and birth outcomes. Higher neighborhood deprivation has been associated with poor birth outcomes, particularly for non-Hispanic White women [28], while residential racial segregation has been associated with low birthweight for non-Hispanic black women [29]. In a systematic review by Blumenshine et al. [6], area-level factors, such as unemployment and poverty concentration, were associated with poor birth outcomes across race/ethnic groups. In the US, income, specifically, was most consistently associated with low birthweight, preterm birth and small for gestational age, though, in contrast with our study, there were no significant racial differences [6].

For women in lower income categories, one theory that can account for the variation in abdominal circumference by income observed in our study stems from endogenous energy storage. The liver stores glucose and both visceral and peripheral fat stores are located within the abdomen as sources of energy [30]. Using income as a proxy for access and subsequent nutrition, those with limited means may have increased fetal consumption of fat stores, resulting in a decrease in the abdominal circumference [31].

The major strengths of our study include the racial/ethnic diversity and the low risk characteristics of our cohort, which allowed us to limit confounding factors such as chronic medical diseases or lifestyle behaviors including smoking that may have clouded the association between socioeconomic factors and fetal growth. Previous studies have analyzed birthweight as a potential adverse outcome in higher risk populations, including mothers with a higher body mass index, cigarette use, and preterm deliveries [25,32,33]. Detailed neonatal anthropometry, however, following a standardized protocol, helps to better elucidate the difference in birthweight as it relates to socioeconomic factors. Anthropometric measurements better characterize neonatal body composition compared to birthweight alone, and has greater relevance to clinical care, as neonatal adiposity rebound is predictive of later metabolic health, while neonatal lean mass is predictive of later cognitive function [12,34]. Furthermore, specific body types, particularly “thin fat” which reflects a small abdominal circumference and low muscle mass, can be associated with an increased predisposition to medical conditions such as type two diabetes mellitus [35]. While our study did not include muscle mass in its anthropometric measurements, the clinical implications of these measurements are evident.

One potential limitation to our study may be the unequal distribution of women in income categories across race/ethnic groups. Specifically, only 10% of non-Hispanic white women reported an annual income of less than $50,000 per year, which may contribute to why we did not observe anthropometric differences by income within this group. Another limitation is the inability to evaluate area-level socioeconomic factors, such as poverty, or to tease apart the nuances of individual socioeconomic factors, such as accrued wealth vs. annual household income. Specifically, there is a history of inequity in accrual of wealth in the United States, and current household income may not be an adequate proxy to explain racial/ethnic differences observed in wealth accumulation across generations [36].

We did not find an impact of individual socioeconomic factors on the association between race/ethnicity and fetal growth, but this finding does not rule out disparities that may be caused by more widespread, societal factors, like poverty or chronic stress associated with racism. By selecting parameters including income, education, marital status, and insurance status, we assumed that, in aggregate, socioeconomic impact could be adequately assessed. Recently, review articles have discussed the perils in limiting our approach to socioeconomic status in health to a one-dimensional analysis [28,29]. Of all the socioeconomic factors evaluated, family income and marital status were statistically significant for all neonatal anthropometric measurements. However, once race and ethnicity were added to the analysis, the effect of family income was attenuated. There was only a difference in abdominal circumference across income as it related to race. This finding suggests that some race/ethnic differences in abdominal circumference may be associated with income, consistent with previous discussions on the interaction between socioeconomic factors and race/ethnicity [37].

The difficulty in trying to understand the socioeconomic impact on outcomes such as neonatal anthropometric measurements across race is in discerning the contextual framework of one’s socioeconomic status. Specifically, life course theories describe how experience across a woman’s lifetime may have a greater influence on birth outcomes compared with those around the time of childbirth [33,38,39]. One longitudinal study described differences in health outcomes among poor black, Puerto Ricans and whites of the same income, and found that disparities in health outcomes of blacks and Puerto Ricans relative to their white counterparts could be explained by the concentration of high poverty in the neighborhoods in which blacks and Puerto Ricans reside [32].

There is substantial evidence supporting the differences in fetal growth across racial and ethnic groups. Understanding these differences has been challenging given that only 15% of this variation may be explained by genetic factors [40]. Studies on birthweight and nativity have found that foreign-born African women are at lower risk of low birthweight compared with US-born African Americans, suggesting that race/ethnic disparities in birthweight may be due to environmental rather than genetic factors [41]. Recently, it has been demonstrated that Africans have higher birthweight-lowering genetic variants than Europeans [42], further supporting the notion that racial/ethnic variation is most likely explained by a combination of genetic and environmental risk factors, such as individual and socioeconomic structural factors.

Socioeconomic factors are associated with overall differences in fetal growth, but not with race/ethnic differences in fetal growth. This finding poses several challenges. The first is revisiting our approach to exploring the impact of socioeconomic status in health outcome research. Our analysis was limited to individual level socioeconomic factors that reflect a moment in time, i.e., one’s gestational period, understanding that the effects of socioeconomic factors have far reaching and lasting effects on one’s development. It is beyond the scope of this investigation to test multi-level models of area-level socioeconomic factors, but our results support current evidence suggesting that individual-level socioeconomic factors alone are unlikely to explain racial and ethnic disparities in birthweight and fetal growth.

Thus, our approach to analyzing the socioeconomic impact on health outcomes may not entirely reflect the SES impact in totality. By expanding our cohort to include a larger sample reflective of different population settlements across the country, i.e., rural, suburban, and urban regions, and by considering structural factors that influence the contextual experience of SES over time, we can begin to unravel the nuances of how socioeconomic factors are linked to health outcomes. This may serve as the missing link between these two dyads, namely racial/ethnic differences among birth outcomes and socioeconomic factors’ influence on birth outcomes.

## 5. Conclusions

Individual socioeconomic factors did not account for the observed differences in fetal growth as measured by birthweight and neonatal anthropometrics amongst racial and ethnic groups. A more expansive look at how socioeconomic and other factors indirectly shape access and subsequently health outcomes such as fetal growth is needed.

## Figures and Tables

**Table 1:** Maternal and neonatal characteristics among self-reported racial/ethnic groups.

Characteristic	Non-Hispanic White	Non-Hispanic Black	Hispanic	Asian/Pacific Islander	Overall
	*n* = 468	*n* = 401	*n* = 454	*n* = 322	*n* = 1645
Maternal age, years—mean ± (SD)	30.3 (4.3)	25.4 (5.3)	27.0 (5.4)	30.5 (4.4)	28.2 (5.3)
Height, cm—mean ± (SD)	165.7 (7.1)	164.4 (6.8)	159.9 (6.2)	160.3 (5.9)	162.7 (7.0)
Pregravid Weight, kg—mean ± (SD)	63.5 (9.0)	64.9 (9.6)	62.3 (9.1)	56.9 (8.2)	62.2 (9.5)
Full-time employment/student status, *n* (%)					
No	79 (16.9%)	101 (25.2%)	178 (39.2%)	103 (32.0%)	461 (28.0%)
Yes	389 (83.1%)	300 (74.8%)	276 (60.8%)	219 (68.0%)	1184 (72.0%)
Marital status ^a^, *n* (%)					
Not married	27 (5.8%)	207 (51.8%)	120 (26.4%)	28 (8.7%)	382 (23.3%)
Married or living with partner	440 (94.2%)	193 (48.3%)	334 (73.6%)	294 (91.3%)	1261 (76.7%)
Insurance, *n* (%)					
Other	25 (5.3%)	199 (49.6%)	275 (60.6%)	49 (15.2%)	548 (33.3%)
Private or managed care	443 (94.7%)	202 (50.4%)	179 (39.4%)	273 (84.8%)	1097 (66.7%)
Family income ^a^, *n* (%)					
<$30,000	17 (3.8%)	168 (48.3%)	144 (38.2%)	40 (16.5%)	369 (26.0%)
$30,000–$39,999	14 (3.1%)	26 (7.5%)	64 (17.0%)	15 (6.2%)	119 (8.4%)
$40,000–$49,999	15 (3.3%)	43 (12.4%)	40 (10.6%)	15 (6.2%)	113 (8.0%)
$50,000–$74,999	58 (12.8%)	31 (8.9%)	52 (13.8%)	32 (13.2%)	173 (12.2%)
$75,000–$99,999	86 (19.0%)	34 (9.8%)	29 (7.7%)	52 (21.4%)	201 (14.1%)
$100,000 or more	263 (58.1%)	46 (13.2%)	48 (12.7%)	89 (36.6%)	446 (31.4%)
Education, *n* (%)					
<High school	4 (0.9%)	44 (11.0%)	99 (21.8%)	17 (5.3%)	164 (10.0%)
High school/equivalent	22 (4.7%)	114 (28.4%)	108 (23.8%)	38 (11.8%)	282 (17.1%)
Some college/associate	87 (18.6%)	144 (35.9%)	169 (37.2%)	61 (18.9%)	461 (28.0%)
Bachelor’s degree	192 (41.0%)	63 (15.7%)	62 (13.7%)	102 (31.7%)	419 (25.5%)
Postgraduate degree	163 (34.8%)	36 (9.0%)	16 (3.5%)	104 (32.3%)	319 (19.4%)
Parity, *n* (%)					
0	249 (53.2%)	196 (48.9%)	173 (38.1%)	164 (50.9%)	782 (47.5%)
1	159 (34.0%)	135 (33.7%)	173 (38.1%)	125 (38.8%)	592 (36.0%)
≥2	60 (12.8%)	70 (17.5%)	108 (23.8%)	33 (10.2%)	271 (16.5%)
Gestational age at delivery—weeks, mean (S.D.)	39.6 (1.0)	39.5 (1.0)	39.6 (1.0)	39.4 (1.1)	39.5 (1.0)
Infant sex, *n* (%)					
Male	255 (54.5%)	200 (49.9%)	225 (49.6%)	165 (51.2%)	845 (51.4%)
Female	213 (45.5%)	201 (50.1%)	229 (50.4%)	157 (48.8%)	800 (48.6%)

^a^ Not included in the totals are missing data: marital status (*n* = 2) and income (*n* = 224: 15 from Non-Hispanic white, 53 from Non-Hispanic black, 77 from Hispanic, 79 from Asian and Pacific Islander). Note: percentages may not add up to 100% due to rounding. All characteristic comparisons among racial/ethnic groups were statistically significant at *p <* 0.0001 for χ^2^ tests for categorical data and ANOVA for continuous data except for infant sex.

**Table 2:** Means and standard deviations for neonatal anthropometry measures by race/ethnicity.

Neonatal Anthropometry (Units)	Non-Hispanic White		Non-Hispanic Black		Hispanic		Asian/Pacific Islander		Overall		*p*-Value	Variable *n*
	*n* = 468		*n* = 401		*n* = 454		*n* = 322		*n* = 1645			
	mean	SD	mean	SD	mean	SD	mean	SD	mean	SD		
Birthweight (gm)	3498	425	3273	415	3382	422	3325	414	3377	428	<0.0001	1644
Exam Weight (gm)	3436	413	3229	414	3320	420	3265	413	3320	423	<0.0001	1642
Exam Length (cm)	50.7	2.5	49.9	2.2	50.2	2.3	50.3	2.4	50.3	2.4	<0.001	1632
Exam Head Circumference (cm)	34.4	1.4	33.8	1.4	34.2	1.3	34.1	1.4	34.1	1.4	<0.0001	1643
Abdominal Circumference (cm)	33.7	1.9	32.3	2.2	33.4	1.9	33.1	2.2	33.2	2.1	<0.0001	1637

Note: All one-way ANCOVAs with race/ethnicity as the independent variable adjusting for full-time employment/student status, marital status, health insurance source, income, education, days from birth (except birthweight), infant sex, and maternal characteristics: age, height, pre-gravid weight, and parity were statistically significant at *p* < 0.001. Type III sums of squares *p*-values for race/ethnicity are presented. The analysis used imputed data.

**Table 3:** Means and SDs for neonatal anthropometric measurements by each category of socioeconomic factor.

	Birthweight (gm)	*p*-Value	Exam Weight (gm)	*p*-Value	Exam Length (cm)	*p*-Value	Exam Head Circumference (cm)	*p*-Value	Exam Abdominal Circumference (cm)	*p*-Value
Full-time employment/student status, mean ± (SD)		0.20		0.20		0.38		0.24		0.54
No (*n* = 461)	3355 (433)		3298 (432)		50.2 (2.3)		34.1 (1.5)		33.2 (2.1)	
Yes (*n* = 1184)	3386 (426)		3329 (419)		50.3 (2.4)		34.2 (1.4)		33.2 (2.1)	
Marital status, mean ± (SD)		<0.0001		0.0002		0.0001		0.003		<0.0001
Not married (*n* = 382)	3300 (431)		3250 (425)		49.9 (2.3)		33.9 (1.5)		32.6 (2.3)	
Married or living with partner (*n* = 1261)	3400 (425)		3342 (420)		50.4 (2.4)		34.2 (1.4)		33.3 (2.0)	
Insurance, mean ± (SD)		0.001		0.003		0.02		<0.0001		0.003
Other (*n* = 548)	3323 (427)		3274 (423)		50.1 (2.3)		33.9 (1.3)		33.0 (2.2)	
Private or managed care (*n* = 1097)	3404 (426)		3343 (421)		50.4 (2.4)		34.2 (1.4)		33.3 (2.0)	
Family income, mean ± (SD)		0.0002		0.001		0.01		0.001		0.0002
<$30,000 (*n* = 369)	3335 (418)		3282 (413)		50.1 (2.4)		34.0 (1.3)		32.9 (2.2)	
$30,000–$39,999 (*n* = 119)	3302 (428)		3245 (417)		49.9 (2.2)		34.0 (1.2)		33.1 (2.1)	
$40,000–$49,999 (*n* = 113)	3351 (409)		3305 (411)		50.3 (2.2)		33.8 (1.6)		33.0 (2.2)	
$50,000–$74,999 (*n* = 173)	3417 (452)		3351 (461)		50.5 (2.2)		34.2 (1.5)		33.2 (2.0)	
$75,000–$99,999 (*n* = 201)	3444 (412)		3380 (411)		50.7 (2.4)		34.3 (1.3)		33.5 (1.9)	
$100,000 or more (*n* = 446)	3430 (426)		3367 (415)		50.4 (2.4)		34.3 (1.4)		33.4 (2.1)	
Education, mean ± (SD)		0.0003		0.001		0.0001		0.001		0.05
<High school (*n* = 164)	3294 (416)		3255 (411)		49.8 (2.1)		33.8 (1.3)		33.0 (2.1)	
High school/equivalent (*n* = 282)	3331 (419)		3271 (412)		50.1 (2.3)		34.1 (1.5)		33.0 (2.1)	
Some college/associate (*n* = 461)	3374 (442)		3311 (435)		50.2 (2.5)		34.1 (1.4)		33.1 (2.2)	
Bachelor’s degree (*n* = 419)	3416 (436)		3359 (429)		50.4 (2.3)		34.3 (1.3)		33.3 (2.0)	
Postgraduate degree (*n* = 319)	3417 (402)		3358 (403)		50.7 (2.5)		34.2 (1.4)		33.3 (2.0)	

Note: *p*-values are for the Wald chi-squared from logistic regression models adjusted for days to exam (except for birthweight).

**Table 4:** Type III sum of squares p-values for race by socioeconomic interactions.

Neonatal Anthropometry (Units)	Full-Time Employed/Student *p*-Value	Marital Status *p*-Value	Insurance *p*-Value	Income *p*-Value	Education *p*-Value	Infant Sex *p*-Value	Age *p*-Value	Height *p*-Value	Weight *p*-Value	Parity *p*-Value
Birthweight (gm)	0.83	0.13	0.46	0.29	0.13	0.52	0.51	0.52	0.65	0.33
Exam Weight (gm)	0.83	0.19	0.70	0.33	0.23	0.43	0.46	0.62	0.81	0.19
Exam Length (cm)	0.99	0.11	0.62	0.46	0.42	**0.03**	0.38	0.59	0.66	0.83
Exam Head circ. (cm)	0.55	0.51	0.99	0.48	0.33	**0.03**	0.31	0.48	0.79	0.55
Abdominal Circumference (cm)	0.69	0.05	0.63	**0.03**	0.26	0.62	0.92	0.70	0.41	0.24

Note: Results are for two-way ANCOVA models adjusted for maternal characteristics, days to exam and infant sex. Missing values for Marital Status (*n* = 2) and Income (*n* = 224) were imputed. Bold text indicates a statistically significant interaction.
